# Pigment Epithelium-Derived Factor (PEDF) Protects Osteoblastic Cell Line from Glucocorticoid-Induced Apoptosis via PEDF-R

**DOI:** 10.3390/ijms17050730

**Published:** 2016-05-13

**Authors:** Shengcheng Yao, Yingnan Zhang, Xiaoyu Wang, Fengchao Zhao, Maji Sun, Xin Zheng, Hongyan Dong, Kaijin Guo

**Affiliations:** 1 Department of Orthopaedic Surgery, Affiliated Hospital of Xuzhou Medical University, Xuzhou 221006, China; surgeony@126.com (S.Y.); Zhjianzh@sina.com (F.Z.); holycityy@126.com (M.S.); thindy1980@163.com (X.Z.); 2 Department of Oncology, Affiliated Hospital of Xuzhou Medical University, Xuzhou 221006, China; zyn_xzmc@163.com; 3 Department of Thoracic Cardiovascular Surgery, Affiliated Hospital of Xuzhou Medical University, Xuzhou 221006, China; wxy_xzmc@163.com; 4 Research Facility Center for Morphology, Xuzhou Medical University, Xuzhou 221004, China; dhy@xzmc.edu.cn

**Keywords:** pigment epithelium-derived factor, glucocorticoids, apoptosis, pigment epithelium-derived factor receptor, lysophosphatidic acid, PI3K/Akt signaling

## Abstract

Pigment epithelial-derived factor (PEDF) is known as a widely expressed multifunctional secreted glycoprotein whose biological actions are cell-type dependent. Recent studies demonstrated that PEDF displays cytoprotective activity in several cell types. However, it remains unknown whether PEDF is involved in glucocorticoid-induced osteoblast death. The aim of this study was to examine the role of PEDF in osteoblast survival in response to dexamethasone, an active glucocorticoid analogue, and explore the underlying mechanism. In the present study, dexamethasone (DEX) was used to induce MC3T3-E1 pre-osteoblast apoptosis. PEDF mRNA and protein levels and cell apoptosis were determined respectively. Then PEDF receptor (PEDF-R)- and lysophosphatidic acid (LPA)-related signal transductions were assessed. Here we show that DEX down-regulates PEDF expression, which contributes to osteoblast apoptosis. As a result, exogenous recombinant PEDF (rPEDF) inhibited DEX-induced cell apoptosis. We confirmed that PEDF-R was expressed on MC3T3-E1 pre-osteoblast membrane and could bind to PEDF which increased the level of LPA and activated the phosphorylation of Akt. Our results suggest that PEDF attenuated DEX-induced apoptosis in MC3T3-E1 pre-osteoblasts through LPA-dependent Akt activation via PEDF-R.

## 1. Introduction

Glucocorticoids (GCs) are frequently used for the management of inflammation and autoimmune disorders. However, prolonged and/or overdosed GC therapy is the leading cause of secondary osteoporosis, and an important contributor in osteonecrosis development [1]. Among the severe acute respiratory syndrome patients, approximately 39% develop osteonecrosis within a few months of GC treatment [2]. Although previous studies reported changes in the bone microenvironment [3], the underlying molecular mechanisms of GC-induced side effects are still not fully understood.

Bone remodeling regulates the bone metabolism through the maintenance of the balance between resorption and formation. Previous studies found that GCs could inhibit bone marrow-derived mesenchymal stem cell proliferation, promote adipogenic differentiation and induce osteoblast apoptosis [4,5,6], thus breaking the balance between bone resorption and formation. The majority of GC effects depend greatly on the glucocorticoid receptor (GR). GR is involved in multiple signaling pathways and is critical for both cell survival and cell death [7,8]. Evidence has shown that GR is widely expressed in many cell types, including osteoblasts and osteoclasts [7,9]. However, the molecular mechanisms underlying GC-induced apoptosis remain undefined, hindering the treatment and prevention of GC-induced adverse effects on the skeleton.

Recent studies have shown that pigment epithelial-derived factor (PEDF) is expressed by bone cells, particularly at the sites of active bone formation [10], indicating the important role of PEDF in bone physiology and pathophysiology. However, PEDF involvement in GC-induced osteoblast apoptosis and whether GCs affect PEDF expression in osteoblasts have not been reported. PEDF is a secreted glycoprotein of roughly 50 kDa, with 418 amino acids. Although first identified in cultured pigment epithelial cells from fetal human retinas, PEDF is widely expressed throughout the human body, with multiple biological functions [11,12]. Furthermore, recent studies revealed that PEDF displays cytoprotective activity in several cell types such as granule cells, photoreceptor cells and cardiomyocytes [13,14,15]. In bone, PEDF induces the differentiation of mesenchymal stem cells (MSCs) and osteoblast precursor cells, which contributes to bone formation [16]. Broadhead *et al.* [17] reported that PEDF expression plays a key role in endochondral ossification, which is essential for bone growth and healing.

PEDF effects are blocked by antibodies which are cell surface-binding antagonists, implying that these cells contain PEDF receptor molecules available to interact with PEDF [18,19]. An 80 kDa PEDF receptor is a member of PNPLA2 (patatin-like phospholipase domain-containing 2), and also known as adipocyte triglyceride lipase (ATGL) which transports protein-2.2, Ca^2+^ independent phospholipase A2 and desnutrin [20]. Upon PEDF binding, PEDF receptor (PEDF-R) induces phospholipase A2, liberating lysophosphatidic acid (LPA) and fatty acids that are involved in a variety of cellular activities [18,21,22]. The Akt pathway is able to integrate stimuli from a variety of signals initiated by hormone/growth factor signaling, and mechanical loading. Phospholipids and LPA have recently emerged as bioactive compounds that exert mitogenic effects and display cytoprotective activities through the stimulation of Akt phosphorylation in many cell types, including osteoblasts [23]. As GCs and PEDF have major effects on the skeletal system, it is of great importance to understand the effects of dexamethasone (DEX) on PEDF expression, as well as the PEDF involvement in DEX-induced osteoblast apoptosis.

## 2. Results

### 2.1. Dexamethasone Induces Apoptosis in MC3T3-E1 Pre-Osteoblasts

**Figure 1:**
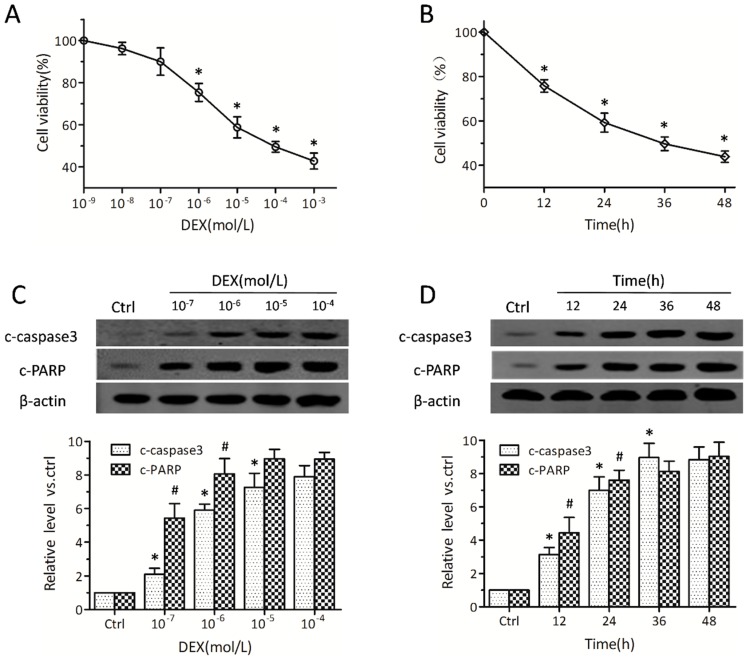
Dexamethasone (DEX) induces cell death and promotes apoptosis in MC3T3-E1 pre-osteoblasts. MC3T3-E1 pre-osteoblasts were exposed to DEX at different concentrations (10^−9^, 10^−8^, ···, 10^−3^ mol/L) for 24 h; in addition, cells were incubated with 10^−5^ mol/L DEX for different times (0, 12, 24, 36, and 48 h). (**A**,**B**) Cell viabilities were detected by CCK-8 (cell counting kit-8) (*n* = 4, * *p* < 0.05 *vs.* the two adjacent groups); (**C**,**D**) Cleaved caspase-3 and poly(ADP-ribose) polymerase (PARP) amounts were assessed by Western blot (*n* = 4, * *p* and # *p* < 0.05 *vs.* the two adjacent groups). Data were expressed in fold induction, relative to baseline or control values. Data are mean ± SD.

Initially, we determined whether osteoblasts undergo cell death upon treatment with DEX. MC3T3-E1 pre-osteoblasts were exposed to a wide range of DEX concentrations for 24 h; in addition, 10^−5^ mol/L DEX was assessed at different time points. According to CCK-8 (cell counting kit-8) assay (Figure 1A,B), cell death rates were increased with doses and prolonged DEX treatments, in agreement with data obtained for primary osteoblasts and other osteoblastic cell lines [5]. DEX treatment caused approximately 40%–50% cell death at 10^−5^ mol/L for 24 h (Figure 1A,B); this concentration was used in subsequent experiments. Correspondingly, Western blot showed a stark elevation in caspase-3, which was markedly activated within 12 h in osteoblasts treated with DEX (Figure 1D). The levels of poly (ADP-ribose) polymerase (PARP), which also reflects apoptosis, were increased in a similar fashion after DEX treatment as shown (Figure 1C,D). No obvious difference was observed in caspase-3 activity at 36 h or more. These results indicated that DEX promoted MC3T3-E1 cell death by activating apoptosis-related signaling.

### 2.2. Dexamethasone Regulates Pigment Epithelial-Derived Factor (PEDF) at the Gene and Protein Levels

**Figure 2:**
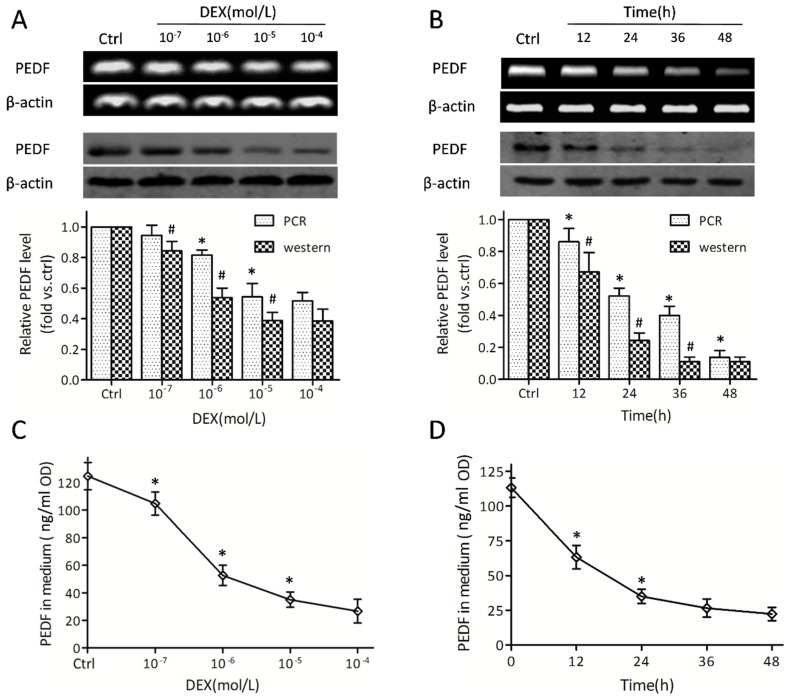
Dexamethasone down-regulates pigment epithelial-derived factor (PEDF) in MC3T3-E1 pre-osteoblasts. MC3T3-E1 pre-osteoblasts were exposed to DEX at different concentrations (10^−7^, 10^−6^, 10^−5^, and 10^−4^ mol/L) for 24 h; in addition, cells were incubated with 10^−5^ mol/L DEX for different times (0, 12, 24, 36, and 48 h). (**A**,**B**) PEDF mRNA levels (**upper** panels) and protein levels (**lower** panels) were detected by RT-PCR and Western blot, respectively (*n* = 5, * *p* and # *p* < 0.05 *vs.* the two adjacent groups). Data were expressed in fold induction, relative to control; (**C**,**D**) The PEDF protein secreted into the medium was tested by ELISA, with results normalized to the corresponding protein concentration (*n* = 5, * *p* < 0.05 *vs.* the two adjacent groups). Data are mean ± SD.

Then we investigated the transcription modulating role of DEX on PEDF. Interestingly, a decrease of PEDF on mRNA and PEDF protein levels, respectively, was observed (Figure 2A,B). A more obvious change was observed in PEDF protein amounts compared with mRNA levels, at the same doses and time points. After incubating in the presence of 10^−5^ mol/L DEX for 24 h, an approximate 70% reduction in relative PEDF protein was observed (Figure 2A,B). Since PEDF is a secreted glycoprotein, PEDF protein levels were also assessed in the culture medium. DEX showed a highly suppressing effect on PEDF protein synthesis (Figure 2C,D). Enzyme linked immunosorbent assay (ELISA) analysis showed that PEDF in supernatants was markedly attenuated with prolonged DEX treatments (Figure 2D). These results suggested that MC3T3-E1 pre-osteoblasts expressed and secreted PEDF, while DEX reduced both PEDF expression and secretion.

### 2.3. PEDF Binds to PEDF receptor (PEDF-R) on the Osteoblast Membrane

**Figure 3:**
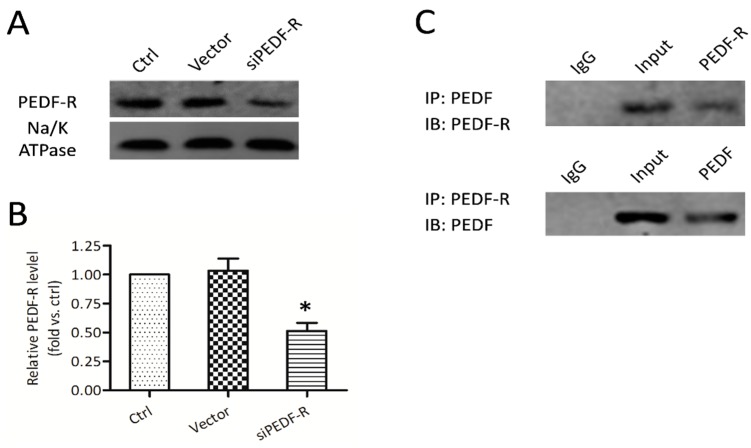
Expression and interaction of PEDF and PEDF receptor (PEDF-R) in MC3T3-E1 cells. RNA interference assays were utilized to knockdown PEDF-R. (**A**) PEDF-R expression detected from membrane protein extracts of MC3T3-E1 pre-osteoblasts by Western blot; (**B**) Results were presented as fold induction, relative to control. (*n* = 4, * *p* < 0.05); (**C**) After 12 h treatment with recombinant PEDF (rPEDF) (10 nmol/L), membrane protein extracts of MC3T3-E1 cells were immunoprecipitated with PEDF or PEDF-R antibodies, the precipitates were detected using PEDF or PEDF-R antibodies.

Membrane proteins were extracted from crude cell lysates as described previously. PEDF-R and Na/K ATPase in membrane fractions were detected. To determine whether PEDF exerts its biological functions through PEDF-R, we silenced PEDF-R (Figure 3A). Interestingly, siRNA-mediated PEDF-R knockdown significantly suppressed PEDF-R protein expression in MC3T3-E1 cells by 40% (Figure 3B). No significant changes were observed in the vector group. After 24 h treatment with recombinant PEDF (rPEDF), PEDF co-immunoprecipitated with PEDF-R was obtained in membrane fractions using anti-PEDF antibodies (Figure 3C). A similar result was observed when anti-PEDF-R antibodies were used (Figure 3C), indicating a direct interaction between PEDF and PEDF-R.

### 2.4. PEDF Attenuates DEX-Induced MC3T3-E1 Pre-Osteoblast Apoptosis via PEDF-R

**Figure 4:**
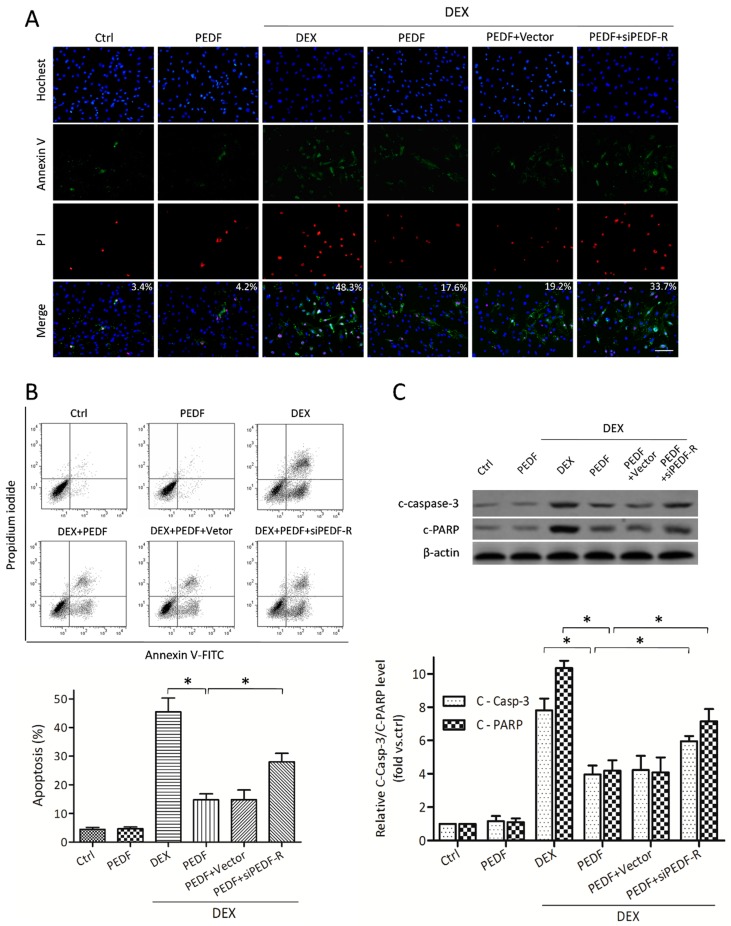
The effect of PEDF on DEX-induced MC3T3-E1 pre-osteoblast apoptosis. MC3T3-E1 cells treated with or without rPEDF (10 nmol/L) were exposed to 10^−5^ mol/L DEX for 24 h. (**A**) Annexin V/PI and Hochest33342 staining assessed by fluorescence microscopy was performed to evaluate apoptosis (Annexin V+/Hochest+, mean percentage from 20 observation fields, bar = 200 μm); (**B**) FACS was also employed to quantify Annexin V/PI staining for apoptosis assessment. Results are expressed as apoptosis percentages (Annexin V+/Hochest+, *n* = 4, * *p* < 0.05); (**C**) Western blot showing the levels of cleaved caspase-3 and PARP (*n* = 5, * *p* < 0.05). Results were presented as fold induction, relative to control. Data are mean ± SD.

RNA interference assays were utilized to knock down PEDF-R and evaluate PEDF/PEDF-R interaction. When treated with DEX, osteoblasts exhibited phosphatidylserine externalization as determined by significantly increased Annexin levels and decreased plasma membrane integrity, as shown by propidium iodide fluorescence (Figure 4A). PEDF attenuated DEX-induced MC3T3-E1 pre-osteoblast apoptosis, while PEDF-R knockdown increased apoptotic cells number (Figure 4B). No significant changes in apoptosis were observed after rPEDF treatment under normal conditions. After treatment with DEX, caspase-3 activity was increased in osteoblasts. Treatment with rPEDF significantly inhibited the levels of caspase-3 and PARP, while this effect was weakened by siPEDF-R (Figure 4C). These findings demonstrated that MC3T3-E1 pre-osteoblasts with reduced PEDF expression exhibited increased apoptosis compared with controls, an effect restored by supplementation with exogenous PEDF protein. The protective effect of PEDF reducing DEX-induced MC3T3-E1 apoptosis was attenuated by PEDF-R siRNA, suggesting that PEDF protected against DEX-induced MC3T3-E1 pre-osteoblast apoptosis by partly interacting with PEDF-R.

### 2.5. PEDF-R Mediates PEDF-Increased Lysophosphatidic Acid (LPA) Levels, Activating Phosphatidylinositol 3-Kinase (PI3K)/Akt Signaling in MC3T3-E1 Pre-Osteoblasts

**Figure 5:**
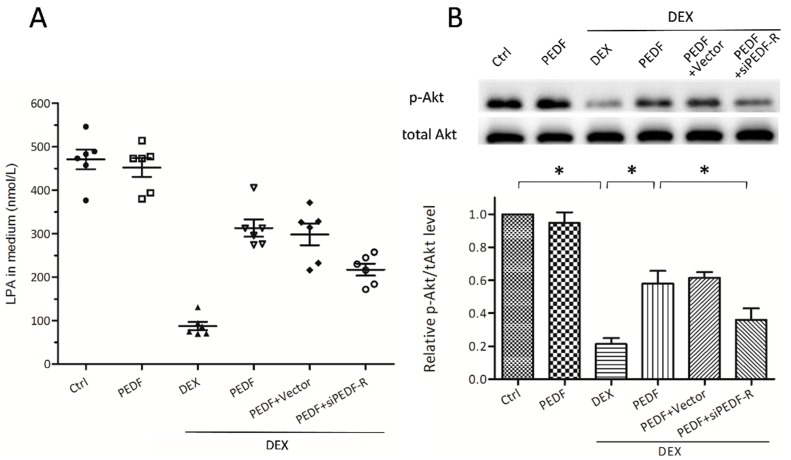
PEDF-R mediated PEDF activates PI3K/Akt pathway by increasing LPA levels in MC3T3-E1 pre-osteoblasts. MC3T3-E1 pre-osteoblasts treated with or without rPEDF were exposed to 10^−5^ M DEX for 24 h. RNA interference assays were performed to silence PEDF-R. (**A**) LPA levels in the medium were tested by ELISA, with results normalized to the corresponding protein concentration (*n* = 6, * *p* < 0.05); (**B**) pAkt (phosphorylated Akt) and tAkt (total Akt) amounts were detected by Western blot. The ratio of pAkt/tAkt were expressed as fold induction (*n* = 5, * *p* < 0.05), relative to control. Data are mean ± SD.

The phospholipase activity of PEDF-R is essential for the production of lysophosphatidic acid (LPA), which stimulates phosphatidylinositol 3-kinase (PI3K)/Akt signaling, thereby protecting against DEX-induced apoptosis. In this experiment, ELISA analysis showed a significant reduction of LPA after treatment with DEX (Figure 5A). PEDF treatment could mitigate the DEX-induced LPA reduction, while PEDF-R siRNA attenuated this effect. Western blot was used to determine the effect of DEX on phosphorylated Akt (p-Akt). PEDF restrained p-Akt reduction that increased the ratio of pAkt to total Akt (tAkt), thereby activating the downstream protective molecules (Figure 5B).

## 3. Discussion

In the present study, evidence was provided for PEDF-R expression and its essential role in anti-apoptotic effects by interacting with PEDF. More interestingly, we found for the first time that in DEX-induced MC3T3-E1 cell apoptosis, both PEDF mRNA and protein levels were significantly decreased. Here, we demonstrated the importance of PEDF in DEX-induced apoptosis in cultured MC3T3-E1 pre-osteoblasts.

One of the most severe side effects of GC therapy is bone toxicity, which is primarily due to the adverse effects on osteoblasts resulting in osteoporosis and osteonecrosis. Our data confirmed that under DEX treatment, apoptosis pathways are activated. It is generally accepted that GCs could down-regulate several Bcl-2-related (B-cell leukemia/lymphoma 2-related) anti-apoptotic molecules [24,25], highly depending on GR-mediated mechanisms. The translational isoforms of GR could selectively regulate pro-apoptotic enzymes (Granzyme A and caspase-6) in pre-osteoblasts [24], suggesting that GR mediates GC-induced osteoblast apoptosis generally by regulating several specific genes and proteins. Interestingly, we found that both PEDF mRNA and protein levels were significantly decreased after DEX treatment. More convincingly, the amounts of PEDF secreted in the medium were also significantly attenuated. These findings propose a possible way by which DEX initiates MC3T3-E1 pre-osteoblast apoptosis. However, the precise specific molecular mechanism by which GCs influence and regulate PEDF expression remains unclear.

The multifunctionality of PEDF could be explained by the interactions with different cell surface receptors of target cells [19]. Here, we demonstrated that PEDF-R exists on the MC3T3-E1 cell membrane and can bind with PEDF, indicating that MC3T3-E1 pre-osteoblasts are PEDF target cells. Our data confirmed the notion that rPEDF could protect against DEX-induced apoptosis in cultured MC3T3-E1 pre-osteoblasts. Moreover, gene silencing was used to characterize the role of PEDF-R in the PEDF-protective process. Additionally, we have described a similar cytoprotective role of PEDF/PEDF-R in cardiomyocytes [26,27]. Here we found that rPEDF had no effect on MC3T3-E1 pre-osteoblast survival in normal conditions because pro/anti-apoptotic substance levels (e.g., LPA) under normal conditions are relatively stable. However, treatment with DEX down-regulated PEDF expression and modified this balance, subsequently leading to altered PEDF-related signal transduction. Taken together, we speculated that PEDF binding could stimulate PEDF-R activities, generate intracellular signaling and display cytoprotective activity in MC3T3-E1 cells under DEX conditions.

The interaction between PEDF and PEDF-R promotes cell lipolysis, providing insights into potential signaling mechanisms. One of the most important lipolysis products is bioactive LPA, which could bind to its receptors (Gi protein-coupled LPA receptors) and operate as a second messenger that affects cell signaling. LPA is capable of activating the PI3K/Akt pathway and increasing Akt phosphorylation [23,28], which in turn inhibits pro-apoptotic molecules. We observed a change in LPA levels after rPEDF treatment under DEX conditions. The DEX-induced decrease in PEDF expression and lipolysis leads to reduced LPA amounts, which directly affect Akt phosphorylation; exogenous rPEDF increased the production of LPA via PEDF-R, and promoted cell survival. A recent study found that PEDF deficiency in mice results in increased total body fat [21,29], illustrating its systemic role as a negative regulator of adipogenesis. Since PEDF is implicated in lipid metabolism, it may enhance lipolysis to reduce DEX-induced adipogenesis, which in itself is a protective factor. Hence, there is enough data suggesting that PEDF can protect against DEX-induced MC3T3-E1 cell apoptosis and alters cell survival partly through LPA-mediated Akt activation. Meanwhile, osteoblasts undergo apoptosis due in part to PEDF level reduction.

DEX could induce MSC apoptosis, inhibit MSC proliferation and promote adipogenic differentiation [4,30]. MSCs are sensitive to apoptosis with the long-term or high-dose application of DEX [30]. Simultaneously, PEDF is involved in bone remodeling as it affects osteoblastic and osteoclastic differentiation. Recent studies revealed that PEDF up-regulates osteoblastic genes that promote osteoblastic differentiation [31]. Besides, evidence has shown that PEDF is involved in MSC and osteoblast progenitor differentiation; meanwhile, suppression of PEDF leads to decreased alkaline phosphatase levels and mineral deposition [16]. Here, we assessed the relationship between DEX and PEDF, and found that PEDF expression was negatively correlated with DEX-induced MC3T3-E1 pre-osteoblast apoptosis. These results revealed that PEDF displays pro-osteogenesis properties in both osteoblast sources and outcomes. Interestingly, PEDF is reported to impede osteoclastic differentiation by inducing the expression of osteoclast-repressive genes [31]. Akiyama *et al.* [32] found that osteoclast differentiation, RANKL-mediated (receptor activator for nuclear factor-κ B ligand-mediated) survival and bone resorption are inhibited by PEDF. In addition, PEDF-derived peptide 34mer could trigger Wnt signaling, which plays an important role in bone formation and homoeostasis [33]. The existing research, however, suggests that PEDF contributes to osteogenesis and osteoblast survival, providing a potential and effective therapeutic strategy for DEX-induced osteoporosis or other bone-related diseases. Our results that dexamethasone suppresses PEDF expression will provide new insights into dexamethasone function and clinical application.

According to existing research, the PEDF-derived peptide 44mer (Val78–Thr121) is known as the key peptide that interacts with PEDF-R and displays neurotrophic and cytoprotective effects [34,35]. So we speculate that the 44mer peptide has a similar cytoprotective function with the full-length PEDF in the process of GC-induced apoptosis. Compared to full-length PEDF, the 44mer peptide can be more stable and shows weaker immunogenicity. Therefore, we plan to further explore the role of PEDF-derived peptide 44mer, which will offer further possibility for clinical application.

## 4. Materials and Methods

### 4.1. Reagents

The following antibodies were used in this study: Cleaved caspase-3, β-actin antibodies (Cell Signaling Technology, Inc., Danvers, MA, USA). Cleaved PARP antibody (Abcam, Inc., Cambridge, UK). Na/K ATPase, PEDF, ATGL (PEDF-R), phosphorylated Akt (Ser473) and total Akt antibodies (Santa Cruz Biotechnology, Inc., Dallas, TX, USA). Hoechst 33342 and Dexamethasone (DEX) were purchased from Sigma-Aldrich (St. Louis, MO, USA). DEX was dissolved in ethanol and stored at −20 °C. Cell counting kit (CCK-8) was purchased from Dojindo (Kumamoto, Japan). Mem-PER™ Plus Kit (Membrane Protein Extraction Kit) was obtained from Biovision, Inc. (San Francisco, CA, USA). Protein A/G-agarose used in this study was obtained from Thermo Scientific (Waltham, MA, USA). Annexin-V-FLUOS Staining Kit (Roche, Branford, CT, USA) was used to assess apoptosis. ELISA kits for PEDF and lysophosphatidic acid (LPA) were purchased from Cloud-clone Corp. (Houston, TX, USA).

### 4.2. Recombinant Lentivirus Constructs and Viral Production

Recombinant mouse PEDF (rPEDF) was obtained from CUSABIO BIOTECH CO., Ltd. (Wuhan, China). The RNAi vector PEDF-R-RNAi-LV of PEDF-R gene that produces PEDF-R shRNA was successfully constructed and then packaged in 293T cells. The concentrated titer of the virus suspension was 2 × 10^12^ Tu/L. Afterward, transient transfection of MC3T3-E1 cells with siRNA targeting the PEDF-R gene was performed using siRNA Transfection reagents from Santa Cruz, following the manufacturer’s instructions.

### 4.3. Cell Culture

The murine MC3T3-E1 pre-osteoblastic cell line, obtained from American Type Culture Collection (ATCC, Manassas, VA, USA), was used as an osteoblast model. MC3T3-E1 pre-osteoblasts are derived from murine calvaria osteoblasts and have similar behavior to primary calvarial osteoblasts. Cells were maintained in α-MEM medium (Gibco, Waltham, MA, USA) supplemented with 10% fetal bovine serum (Gibco), 100 U/mL penicillin/streptomycin at 37 °C in a humidified atmosphere of 5% CO_2_. DEX was used at different concentrations and times to evaluate the relationship between glucocorticoids and osteoblast apoptosis. After incubation, the assays described below were performed.

### 4.4. Cell Viability Assay

MC3T3-E1 cells were harvested and seeded in 96-well plates at a concentration of 1 × 10^4^ cells/well. After DEX treatment, cell viability was assessed by CCK-8 assay. The absorbance at 450 nm was measured by a microplate reader (BioTek Synergy 2, Winooski, VT, USA). Mean optical density (OD) values from six wells for each group were used to calculate cell viability percentages. Cell culture medium with CCK-8 was used as a negative control.

### 4.5. Western Blot Analysis

For Western blot, cells were lysed in ice-cold lysis buffer (100 mmol/L Tris–HCl, 20% glycerine, 4% sodium dodecyl sulfate, 200 mmol/L dl-Dithiothreitol and protease inhibitors) and the supernatant was collected. The protein collected was then determined using protein reagents (Bio-Rad, Hercules, CA, USA) by the Bradford method. Membrane fractions were obtained with the Mem-PER™ Plus Kit, following the manufacturer’s instructions. Equal amounts of proteins were separated by 7%~12% SDS-PAGE, transferred onto nitrocellulose (NC) membranes (Millipore, Billerica, MA, USA), blocked with 5% non-fat milk in PBS-T for 2 h at room temperature. Then the membranes were immunoblotted with primary antibodies raised against cleaved caspase-3, β-actin (Cell Signaling Technology, Danvers, MA, USA), cleaved PARP (Abcam, Cambridge, UK), PEDF, PEDF-R, phosphorylated Akt (Ser473), total Akt (Santa Cruz Biotechnology, Dallas, TX, USA), and Na/K ATPase (plasma membrane marker), overnight at 4 °C. Fluorescently labeled secondary antibody (Rockland, Limerick, PA, USA) was added for 1 h, and protein bands were scanned by the Infrared Imaging System (Li-Cor Biosciences, Waltham, MA, USA).

### 4.6. Measurement of PEDF mRNA Expression

To assess PEDF mRNA expression, total RNA was extracted using TRIzol Plus RNA Purification Kit (Invitrogen Corporation, Carlsbad, CA, USA), following the manufacturer instructions. RNA was dissolved in 100 μL diethyl pyrocarbonate (DEPC) water. cDNA was synthesized using ScriptRT Reverse Transcriptase (Invitrogen, Carlsbad, CA, USA). Then 1 μg of RNA extracted from each sample was then reverse-transcribed and conventional PCR was conducted. The following primers were used: PEDF-forward (mouse) 5′-CCCTTGACAGGAAGTATGAG-3′ and PEDF-reverse (mouse) 5′-TGCTGAAGTCGGGTGATT-3′; β-actin-forward (mouse) 5′-CCTCTATGCCAACACAGTGC-3′ and β-actin-reverse (mouse) 5′-ACATCTGCTGGAAGGTGGAC-3′. Polymerase chain reaction product band intensities were analyzed using the Quanti Scan software. PCR products were obtained on a DNA thermal cycler, and analyzed by 0.8% agarose gel electrophoresis. Gene expression of PEDF was normalized to β-actin mRNA levels.

### 4.7. Co-Immunoprecipitation

After 24 h treatment with or without PEDF, RNA interference assays were used to knock down PEDF-R. Membrane fractions were obtained by using a Mem-PER™ Plus Kit. Equal amounts of membrane fraction proteins were passed through spin columns with NeutrAvidin gels. The bound biotinylated proteins were eluted by incubating the beads for 60 min in 1× SDS-PAGE sample buffer with 50 mmol/L DTT. Then, the protein sample was pre-cleared using 30 μL of protein A/G-agarose (immobilized) on a rotator at room temperature for 30 min, and centrifuged at 14,000× *g* and 4 °C for 30 min. Then 250 μL of the supernatant was incubated with appropriate antibodies overnight at 4 °C. Protein A-Sepharose CL-4B (50 μL) was then added to the mixture at 4 °C and incubated for another 2 h. The samples were washed with cold immunoprecipitation buffer for three times and then boiled in 4× Laemmli sample buffer for 5 min. After centrifugation (14,000× *g*, 1 min), the supernatant was immunoblotted with appropriate antibodies as described previously in Western blot analysis.

### 4.8. Cell Apoptosis Assessment

After treatment, MC3T3-E1 pre-osteoblasts were harvested and resuspended in PBS at 1 × 10^6^ cells/mL. After centrifugation at 1000 rpm for 4–5 min, the cells were gently resuspended in 500 μL of cold binding buffer, 5 μL of Annexin V-FITC and 5 μL of propidium iodide (PI) under room temperature and dark condition. Following gentle vortexing, samples were analyzed on a flow cytometer (Miltenyi Biotech, Bergisch Gladbach, Germany) within 1 h. Alternatively, MC3T3-E1 cells were also stained with Annexin V-FITC, PI and Hochest33342 *in situ*, and observed on a fluorescence microscope (Olympus, Tokyo, Japan).

### 4.9. Quantification of PEDF and LPA

MC3T3-E1 pre-osteoblasts were incubated in the presence or absence of 10^−5^ mol/L DEX. The supernatants from confluent MC3T3-E1 cells were collected, and lysed in RIPA buffer. Protein concentrations of the cell lysates from each group were determined using protein reagents (Bio-Rad) for the normalization of PEDF and LPA measurements in ELISA. PEDF and LPA levels in the medium were measured using specific ELISA kits (Cloud-clone Corp., Houston, TX, USA), following the manufacturer’s instructions.

### 4.10. Statistics

The values were expressed as the mean ± SD. Statistical analysis of the results was performed using SPSS 19.0 for Windows, by one way ANOVA or repeated-measures analysis of variance followed by the Student-Newman–Keuls test for multiple comparisons. *p* < 0.05 was considered statistically significant.

## 5. Conclusions

Our research has presented a new beneficial role of PEDF in preventing dexamethasone-induced MC3T3-E1 cell apoptosis. This constitutes an important advance in elucidating the molecular mechanisms for PEDFR-mediated cell protection. PEDF may be an important and indispensable factor in stabilizing the bone microenvironment and protecting against GC toxicity. As far as we know, this is the first study to show that GCs down-regulate PEDF expression, which contributes to osteoblast apoptosis. However, the detailed regulatory mechanisms and what happens to PEDF in osteoclasts under DEX treatment need further investigation. This is a primary study that provides a new understanding of the complex PEDF effects on pro-osteogenesis; further *in vivo* assays are planned to confirm these findings.

## Abbreviations

PEDF	Pigment epithelium-derived factor
DEX	Dexamethasone
rPEDF	Recombinant PEDF
PEDF-R	Pigment epithelial-derived factor receptor
GCs	Glucocorticoids
GR	Glucocorticoid receptor
LPA	Lysophosphatidic acid
PNPLA2	Patatin-like phospholipase domain-containing 2
ATGL	Adipose triglyceride lipase
pAkt	Phosphorylated Akt
LBD	Ligand binding domain
